# Reduced genetic influence on childhood obesity in small for gestational age children

**DOI:** 10.1186/1471-2350-14-10

**Published:** 2013-01-22

**Authors:** Dug Yeo Han, Rinki Murphy, Angharad R Morgan, Wen Jiun Lam, John M D Thompson, Clare R Wall, Karen E Waldie, Edwin A Mitchell, Lynnette R Ferguson

**Affiliations:** 1 Discipline of Nutrition, FM&HS, The University of Auckland, Auckland, New Zealand; 2 Department of Paediatrics, FM&HS, The University of Auckland, Auckland, New Zealand; 3 Nutrigenomics New Zealand, Auckland, New Zealand; 4 Department of Medicine, FM&HS, The University of Auckland, Auckland, New Zealand

**Keywords:** BMI, Childhood obesity, AGA children, SGA children

## Abstract

**Background:**

Children born small-for-gestational-age (SGA) are at increased risk of developing obesity and metabolic diseases later in life, a risk which is magnified if followed by accelerated postnatal growth. We investigated whether common gene variants associated with adult obesity were associated with increased postnatal growth, as measured by BMI z-score, in children born SGA and appropriate for gestational age (AGA) in the Auckland Birthweight Collaborative.

**Methods:**

A total of 37 candidate SNPs were genotyped on 547 European children (228 SGA and 319 AGA). Repeated measures of BMI (z-score) were used for assessing obesity status, and results were corrected for multiple testing using the false discovery rate.

**Results:**

SGA children had a lower BMI z-score than non-SGA children at assessment age 3.5, 7 and 11 years. We confirmed 27 variants within 14 obesity risk genes to be individually associated with increasing early childhood BMI, predominantly in those born AGA.

**Conclusions:**

Genetic risk variants are less important in influencing early childhood BMI in those born SGA than in those born AGA, suggesting that non-genetic or environmental factors may be more important in influencing childhood BMI in those born SGA.

## Background

Worldwide more than 30 million infants per year are born small for gestational age (SGA) defined by birth weight below the 10th percentile, according to gestational age [1]. These infants are at increased risk of mortality and morbidity in early life as well as greater later risk of developing obesity, dyslipidemia, hypertension and diabetes.

It has been suggested that growth restriction in the womb resulting from poor maternal nutrition, can program the individual’s physiology and metabolism predisposing them to long-term susceptibility to metabolic diseases [2,3]. Alternative hypotheses explaining the link between low birthweight and higher risk of adult metabolic diseases include the foetal insulin hypothesis [4] that suggests common genetic variants which influence insulin secretion or action may both reduce foetal growth (as insulin is a key foetal growth factor) and increase the risk of type 2 diabetes in later life.

Candidate gene and genome wide association studies have led to the discovery of over 50 gene variants known to be associated with adult obesity [5,6]. Two recent studies involving the Avon Longitudinal Study of Parents and Children [7], and the 1946 British Birth Cohort study [8], have found that a genetic predisposition score of 8 and 11 adult obesity susceptibility alleles were associated with greater infancy and childhood weight, height and BMI gain. The obesity risk allele score showed a weaker association with BMI than with weight until 3.5 years, due to concurrent gains in both length and weight during infancy. While combining established genetic variants for BMI into a risk-allele score maximised statistical power, this approach masked possible heterogeneity in effects of individual variants.

The detrimental effect of poor fetal growth on long-term metabolic health is observed to be magnified if followed by accelerated postnatal growth [9]. The contribution of genetic factors to such accelerated postnatal growth in those born SGA is unknown. We hypothesized that certain obesogenic gene variants would have a disproportionately higher impact in increasing early childhood BMI in those born SGA than those born appropriate for gestational age (AGA), and therefore explain part of the link between low birthweight and subsequent risk of obesity. This is based on the assumption that underlying metabolic programming in those born SGA would enhance the biological metabolic effect of any Mendelian inherited gene variant. We explored the childhood growth of those born SGA and AGA and investigated the association of candidate obesity genes with the BMI z-score in the Auckland Birthweight Collaborative (ABC) study children, in relation to SGA status.

## Methods

### The Auckland birthweight collaborative study

The ABC Study has been described in more detail elsewhere [10]. The study principally investigates factors that relate to childhood growth and development. All children included in this study were born at 37 weeks of gestation or more. Infants were defined as SGA if their birthweight was equal to or below the sex-specific 10th percentile for gestational age in the New Zealand population. Infants were defined as AGA if their birthweight was greater than the sex-specific 10th percentile for gestational age in the New Zealand population.

### Subjects

Eligible for inclusion in the ABC study were all SGA infants and a selection of one in sixteen AGA infants, born in the Waitemata Health or Auckland Healthcare regions in New Zealand between 16 October 1995 and 12 August 1996. All SGA children and a selection of one in eight AGA children, who were born between 12 August 1996 and 30 November 1997 in the Auckland Healthcare region, were also eligible for inclusion. Data have been collected at birth (phase 1), 12 months (phase 2), 3.5 years (phase 3), 7 years (phase 4), and 11 years (phase 5). This study utilises phases 3, 4, and 5 data. A total of 547 children (228 SGA and 319 AGA, phase 5) have completed BMI and 542 children have genotype data. The sample was restricted to children who were born to European mothers because of the poor response rate from non-European ethnic groups at earlier phases. DNA was extracted from the blood/buccal samples using Qiagen’s DNA extraction kit and following the manufacturer’s instructions. The study received ethical approval from the Northern Regional Ethics Committee. The 3.5 year old children’s study had the ethic approval number of 99/097; the study of 7 year old children is AKX/02/00/319; and the study of 11 year old children is NYT/06/00/112. Signed consent for the study was given by the parents of the children at all phases and also given by the child at age 11 years.

### Outcome measure

Body Mass Index (weight/height^2^) is a common method of assessing obesity status that was used in this study. BMI scores were converted to z-scores using the LMS method to ensure normal distribution of the data [11].

### SNP selection

The SNPs were selected from a systematic literature search to identify genetic variants demonstrating association with obesity using the criteria previously described [12]. We included 37 obesity SNPs identified from published candidate gene or genome-wide association studies, located in 21 genes. Genotypic data were not available for nine SNPs: rs1387153 (*MTNR1B*), rs6020339 (*CTNNBL1*), rs2388399 (*PFKP*), rs477181 (*MC4R*), rs3820152 (*ADIPOR1*), rs7561317 (*TMEM18*), rs7647305 (*ETV5 – DGKG*), rs2844479 (*NCR3 - AIF1*), and rs10487818 (*NAMPT*). These SNPs either could not be multiplexed into our sequenom assays, failed or did not pass our quality control measure for inclusion in the analysis. Table 1 provides the list of genes/SNPs selected for investigation including the tested allele and its frequency.

**Table 1:** **Obesity SNPs for association with BMI in the Auckland Birthweight Collaborative study samples (Morgan et al.**[12]**)**

**Gene**	**Name**	**Chr**	**SNP**	**Description**	**Tested allele**	**Tested allele frequency**
ADIPOR2	adiponectin receptor 2	12	rs2286385	Intronic	T	0.339
BDNF	Brain-derived neurotrophic factor	11	rs6265	Missense V66M	G	0.828
BDNF	Brain-derived neurotrophic factor	11	rs925946	9.2 kb upstream	G	0.653
CTNNBL1	catenin (cadherin-associated protein), beta-like 1	20	rs16986921	Intronic	T	0.046
CTNNBL1	catenin (cadherin-associated protein), beta-like 1	20	rs6013029	Intronic	T	0.045
CTNNBL1	catenin (cadherin-associated protein), beta-like 1	20	rs6020395	Intronic	C	0.049
CTNNBL1	catenin (cadherin-associated protein), beta-like 1	20	rs6020712	Intronic	A	0.046
CTNNBL1	catenin (cadherin-associated protein), beta-like 1	20	rs6020846	Intronic	G	0.054
CTNNBL1	catenin (cadherin-associated protein), beta-like 1	20	rs6096781	Intronic	C	0.040
CTNNBL1	catenin (cadherin-associated protein), beta-like 1	20	rs6125962	Intronic	C	0.053
FAIM2	Fas apoptotic inhibitory molecule 2	12	rs7138803	Intergenic	A	0.373
FTO	fat mass and obesity associated gene	16	rs6499640	Intronic	A	0.613
FTO	fat mass and obesity associated gene	16	rs8050136	Intronic	A	0.358
FTO	fat mass and obesity associated gene	16	rs9939609	Intronic	A	0.346
GNPDA2	glucosamine-6-phosphate deaminase 2	4	rs10938397	453.9 kb downstream	G	0.462
HMGA2	high mobility group AT-hook 2	12	rs1042725	UTR-3	C	0.502
INSIG2	insulin-induced gene 2 gene	2	rs2012693	25.8 kb upstream	C	0.607
INSIG2	insulin-induced gene 2 gene	2	rs7566605	10 kb upstream	G	0.678
KCTD15	potassium channel tetramerization domain containing 15	19	rs11084753	17 kb downstream	A	0.347
LPL	lipoprotein lipase	8	rs3200218	3′UTR	G	0.238
MAF	v-maf musculoaponeurotic fibrosarcoma oncogene homolog	16	rs1424233	48 kb downstream	A	0.479
MC4R	melanocortin-4 receptor	18	rs12970134	153.8 kb upstream	A	0.259
MC4R	melanocortin-4 receptor	18	rs17700633	109.1 kb upstream	A	0.309
MC4R	melanocortin-4 receptor	18	rs17782313	187.5 kb upstream	C	0.228
MC4R	melanocortin-4 receptor	18	rs4450508	125.1 kb upstream	A	0.325
MC4R	melanocortin-4 receptor	18	rs502933	142 kb upstream	A	0.317
MTCH2	mitochondrial carrier homolog 2	11	rs10838738	Intronic	G	0.363
MTMR9	myotubularin-related protein 9	8	rs2293855	intronic	A	0.393
NPC1	Niemann-Pick disease, type C1	16	rs1805081	Missense H 215R	A	0.558
NEGR1	Neuronal growth regulator 1	1	rs2568958	16.7 kb downstream	A	0.609
NEGR1	Neuronal growth regulator 1	1	rs2815752	64 kb downstream	T	0.608
PFKP	platelet type phosphofructokinase	10	rs2388395	317 kb upstream	G	0.988
PFKP	platelet type phosphofructokinase	10	rs6602024	Intronic	A	0.106
PTER	phosphotriesterase-related gene	10	rs10508503	179 kb upstream	C	0.917
SEC16B	SEC16 homolog B	1	rs10913469	Intronic	C	0.172
SH2B1	SH2B adaptor protein 1	16	rs7498665	Missense A434T	G	0.614
TMEM18	transmembrane protein 18	2	rs6548238	33 kb upstream	C	0.833

### Genotyping

Genotyping was performed with the MassARRAY and iPlex systems of the Sequenom genotyping platform (Sequenom, San Diego, CA), which uses the MALDI-TOF primer extension assay [13,14], according to manufacturer’s recommendations. Assays were optimized in 24 samples consisting of 20 reference Centre d’Etude du Polymorphisme Humain (CEPH) samples and 4 blanks. All sample plates contained cases, controls, blanks, CEPH and duplicate samples. Quality control measures included independent double genotyping, blind to sample identity and blind to the other caller, and where available, comparison of our CEPH genotypes to those in the HapMap (http://www.hapmap.org).

### Statistical analysis

Generalised linear model was used to test the linearity of genotype-phenotype relationship for quantitative traits. Since the outcomes of interest, BMI z-score, was a repeated measure, the GENMOD procedure with the REPEATED statement in SAS was used to fit a generalised estimating equations (GEE) model. For linearity of the genotype-phenotype relationship for quantitative traits, additive model was used, each SNP was coded 0, 1, and 2 for each tested allele [15]. The association with SNPs was carried out in four ways due to the disproportionate sampling of AGA and SGA at birth (phase 1): using stratified by (1) AGA and (2) SGA children, (3) using a main effect model with adjustment for the SGA status, and (4) interaction with SGA status. False discovery rate (FDR) q-values were calculated for the total number of tests (148 tests; 37 SNPs times four analyses) using the ‘qvalue’ package in R [16,17] with the null hypothesis fraction estimated by the smoothing method of Storey and Tibshirani [18]. A prespecified FDR threshold was set at 0.05. Table 2 provides the results of the analysis of association between SNPs and BMI z-score with both q-values and unadjusted p-values; a SNP is called significant if the q-value is less than the prespecified 0.05. SAS (V9.2 SAS Institute., Cary, NC, USA) and R (R: A language and environment for statistical computing. R Foundation for Statistical Computing, Vienna, Austria. ISBN 3-900051-07-0, http://www.R-project.org) were used for statistical analyses. This resulted in a p-value of 0.05 as being statistically significant.

**Table 2:** Analysis of association between SNPs and repeated measures of BMI z-score

**Gene**	**SNP**	**Tested allele**	**AGA**			**SGA**			**Adjusted for SGA status**			**Interaction with SGA status (ref = AGA)**		
			**Estimate (95% CI)**	**p value**	**q value**	**Estimate (95% CI)**	**p value**	**q value**	**Estimate (95% CI)**	**p value**	**q value**	**Estimate (95% CI)**	**p value**	**q value**
ADIPOR2	rs2286385	T	0.165 (0.065-0.265)	0.0012	0.0073*	0.035 (−0.093-0.162)	0.5950	0.3941	0.110 (0.031-0.190)	0.0063	0.0207*	−0.131 (−0.294-0.031)	0.1134	0.1176
BDNF	rs6265	G	0.123 (0.002-0.244)	0.0470	0.0650	0.203 (0.040-0.365)	0.0146	0.0303*	0.159 (0.059-0.260)	0.0018	0.0095*	0.079 (−0.123-0.282)	0.4429	0.3117
CTNNBL1	rs16986921	T	0.231 (−0.023-0.484)	0.0742	0.0848	0.259 (−0.058-0.576)	0.1089	0.1160	0.245 (0.042-0.449)	0.0180	0.0346*	0.030 (−0.377-0.436)	0.8867	0.4992
CTNNBL1	rs6013029	T	0.295 (0.044-0.547)	0.0214	0.0368*	0.310 (−0.015-0.634)	0.0611	0.0764	0.303 (0.099-0.507)	0.0036	0.0149*	0.016 (−0.396-0.428)	0.9401	0.5099
CTNNBL1	rs6020395	C	0.185 (−0.043-0.413)	0.1108	0.1164	0.458 (0.126-0.791)	0.0069	0.0212*	0.303 (0.109-0.498)	0.0022	0.0108*	0.274 (−0.130-0.677)	0.1836	0.1608
CTNNBL1	rs6020712	A	0.309 (0.050-0.568)	0.0193	0.0357*	0.233 (−0.074-0.539)	0.1372	0.1352	0.270 (0.068-0.472)	0.0087	0.0227	−0.077 (−0.479-0.325)	0.7085	0.4432
CTNNBL1	rs6020846	G	0.219 (−0.008-0.446)	0.0591	0.0751	0.287 (−0.019-0.594)	0.0663	0.0794	0.251 (0.064-0.438)	0.0085	0.0227	0.068 (−0.315-0.452)	0.7265	0.4509
CTNNBL1	rs6096781	C	0.209 (−0.025-0.444)	0.0805	0.0894	0.313 (−0.103-0.729)	0.1408	0.1370	0.249 (0.034-0.464)	0.0232	0.0389	0.103 (−0.376-0.582)	0.6742	0.4320
CTNNBL1	rs6125962	C	0.204 (−0.017-0.424)	0.0700	0.0811	0.276 (−0.029-0.582)	0.0763	0.0859	0.237 (0.054-0.421)	0.0113	0.0241*	0.072 (−0.306-0.450)	0.7076	0.4432
FAIM2	rs7138803	A	0.117 (0.015-0.219)	0.0249	0.0407*	−0.015 (−0.150-0.119)	0.8220	0.4799	0.059 (−0.023-0.141)	0.1609	0.1492	−0.132 (−0.301-0.037)	0.1250	0.1279
FTO	rs6499640	A	0.051 (−0.043-0.145)	0.2905	0.2290	0.153 (0.046-0.260)	0.0050	0.0188*	0.097 (0.027-0.168)	0.0070	0.0212*	0.103 (−0.039-0.246)	0.1562	0.1466
FTO	rs8050136	A	0.143 (0.042-0.243)	0.0055	0.0195*	0.137 (0.011-0.263)	0.0335	0.0489*	0.141 (0.062-0.219)	4.8e-04	0.0042*	−0.005 (−0.166-0.158)	0.9569	0.5130
FTO	rs9939609	A	0.148 (0.048-0.248)	0.0038	0.0150*	0.154 (0.028-0.279)	0.0164	0.0331*	0.300 (0.117-0.482)	1.8e-04	0.0021*	0.007 (−0.154-0.169)	0.9290	0.5099
HMGA2	rs1042725	C	0.104 (0.010-0.198)	0.0304	0.0470*	0.062 (−0.058-0.183)	0.3090	0.2411	0.087 (0.013-0.161)	0.0215	0.0368*	−0.041 (−0.194-0.112)	0.6015	0.3951
KCTD15	rs11084753	A	0.067 (−0.036-0.169)	0.2019	0.1749	0.155 (0.037-0.274)	0.0104	0.0234*	0.103 (0.026-0.181)	0.0090	0.0227*	0.088 (−0.069-0.244)	0.2732	0.2199
MAF	rs1424233	A	0.052(−0.042 -0.146)	0.2758	0.2199	−0.130 (−0.263-0.003)	0.0552	0.0725	−0.024 (−0.103-0.054)	0.5446	0.3669	−0.183 (−0.347- -0.020)	0.0280	0.0441*
MC4R	rs12970134	A	0.290 (0.191-0.389)	1.0e-08	8.0e-07*	−0.060 (−0.204-0.085)	0.4194	0.3061	0.153 (0.069-0.236)	3.3e-04	0.0033*	−0.350 (−0.525- -0.174)	9.6e-05	0.0015*
MC4R	rs17700633	A	0.120 (0.015-0.225)	0.0253	0.0407*	−0.047 (−0.164-0.070)	0.4299	0.3080	0.044 (−0.035-0.123)	0.2762	0.2199	−0.167 (−0.325- -0.010)	0.0374	0.0536
MC4R	rs17782313	C	0.204 (0.105-0.303)	5.6e-05	0.0011*	−0.006 (−0.147-0.136)	0.9398	0.5099	0.122 (0.041-0.204)	0.0032	0.0140*	−0.210 (−0.383- -0.037)	0.0176	0.0346*
MC4R	rs4450508	A	0.189 (0.091-0.286)	1.5e-04	0.0019*	0.021 (−0.115-0.158)	0.7610	0.4622	0.122 (0.041-0.202)	0.0029	0.0134*	−0.168 (−0.336-0.0)	0.0496	0.0674
MC4R	rs502933	A	0.168 (0.070-0.266)	7.5e-04	0.0053*	0.009 (−0.129-0.148)	0.8944	0.4999	0.107 (0.027-0.188)	0.0092	0.0227*	−0.159 (−0.329-0.011)	0.0665	0.0794
MTMR9	rs2293855	A	0.162 (0.063-0.261)	0.0013	0.0073*	−0.039 (−0.156-0.079)	0.5208	0.3538	0.079 (0.003-0.156)	0.0427	0.0601	−0.199 (−0.353- -0.045)	0.0113	0.0241*
NEGR1	rs2568958	A	−0.053 (−0.139-0.033)	0.2280	0.1953	0.135 (0.021-0.250)	0.0203	0.0364*	0.021 (0.048-0.090)	0.5526	0.3691	0.189 (0.046-0.332)	0.0097	0.0227*
NEGR1	rs2815752	T	−0.059 (−0.146-0.028)	0.1810	0.1603	0.133 (0.021-0.245)	0.0195	0.0357*	0.017 (0.052-0.085)	0.6359	0.4108	0.193 (0.052-0.334)	0.0075	0.0214*
PTER	rs10508503	C	0.307 (0.166-0.448)	2.1e-05	8.2e-04*	−0.009 (−0.258-0.240)	0.9446	0.5099	0.190 (0.055-0.324)	0.0057	0.0195*	−0.314 (−0.602- -0.026)	0.0327	0.0489*
SEC16B	rs10913469	C	0.215 (0.089-0.341)	8.4e-04	0.0053*	0.199 (0.048-0.350)	0.0098	0.0227*	0.207 (0.110-0.305)	3.1e-05	8.2e-04*	−0.016 (−0.213-0.181)	0.8759	0.4966
SH2B1	rs7498665	G	−0.100 (−0.191- -0.008)	0.0334	0.0489*	0.163 (0.043-0.283)	0.0076	0.0214*	0.006 (−0.067-0.080)	0.8664	0.4966	0. 263 (0.112-0.414)	6.3e-04	0.0050*

* Remain significant after applying multiple testing correction using false discovery rate.

## Results

### Growth patterns of ABC participants according to SGA status

**Figure 1:**
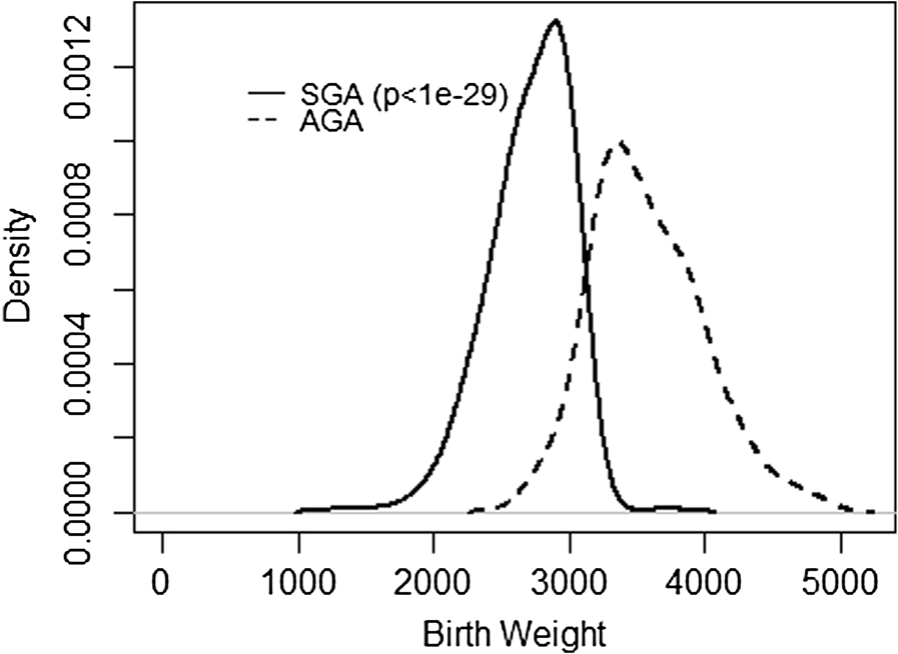
Birth weight by SGA status.

**Figure 2:**
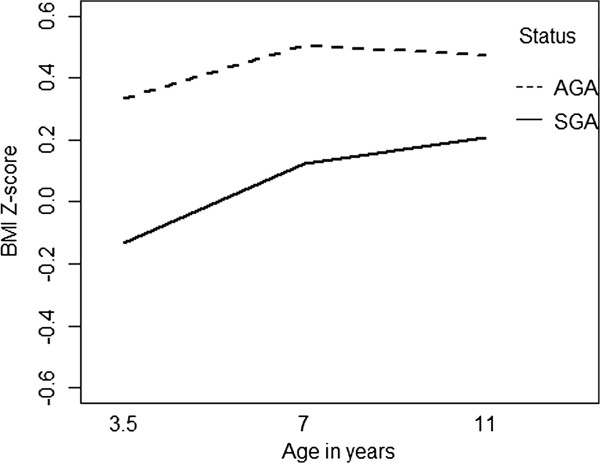
BMI z-score by SGA status.

SGA children had significantly lower BMI z-score than AGA children at all three assessments conducted at 3.5, 7 and 11 years of age (Table 3 and Figure 1). BMI z-score showed no significant interaction between SGA status and age at assessment (p = 0.254) (Table 4 and Figure 2).

**Table 3:** Differences in birth weight and BMI z-score

		**Birth weight (g)**		**BMI z-score**	
		**Estimate (95% CI)**	**p**	**Estimate (95% CI)**	**p**
At Birth	Female	−146.0 (−220.7 – -71.2)	**1.35e-04**		
	Male	0			
	SGA	−896.3 (−959.4 – -833.1)	**<1e-29**		
	AGA	0			
3.5 year old	Female			−0.18 (−0.32 – -0.05)	**0.0075**
	Male			0	
	SGA			−0.46 (−0.60 – -0.33)	**6.26e-11**
	AGA			0	
7 year old	Female			−0.15 (−0.33 – 0.03)	0.0935
	Male			0	
	SGA			−0.38 (−0.56 – -0.20)	**3.88e-05**
	AGA			0	
11 year old	Female			−0.29 (−0.47 – -0.11)	**0.0017**
	Male			0	
	SGA			−0.26 (−0.44 – 0.08)	**0.0058**
	AGA			0	

**Table 4:** BMI z-score Growth by SGA status

	**AGA**		**SGA**		**Adjustment for SGA status**		**Interaction with SGA status**
**Phase**	**Estimate (SE)**	**p**	**Estimate (SE)**	**p**	**Estimate (SE)**	**p**	**p**
At 3.5 year	0		0		0		0.254
At 7 year	0.17 (0.08)	**0.0401**	0.25 (0.11)	**0.0178**	0.20 (0.06)	**0.0018**	
At 11 year	0.14 (0.08)	0.087	0.34 (0.10)	**0.0008**	0.22 (0.06)	**4.03e-04**	

### Genotype data

A total of 37 candidate SNPs for obesity were successfully genotyped (Table 1), and their relationship with BMI z-score was tested. There was no indication of a deviation from Hardy Weinberg equilibrium (HWE), except for one SNP (rs7903146) which was excluded from further analysis. Using a prespecified FDR threshold at α = 0.05 [17], we determined that 27 SNPs were significantly associated with the BMI z-score in at least one of the four analyses: stratified by AGA and SGA children, after adjustment for SGA status, and interaction with SGA status (Table 2).

### Variants associated with BMI z-score in AGA children

A total of 16 variants: rs2286385 (*ADIPOR2*), rs6013029 and rs6020712 (*CTNNBL1*), rs7138803 (*FAIM2*), rs8050136 and rs9939609 (*FTO*), rs1042725 (*HMGA2*), rs12970134, rs17700633, rs17782313, rs4450508, and rs502933 (*MC4R*), rs2293855 (*MTMR9*), rs10508503 (*PTER*), rs10913469 (*SEC16B*), and rs7498665 (*SH2B1*) in 10 genes were found to be significantly associated with BMI z-score in AGA children after correction for multiple testing (Table 2). The impact on BMI z-score increase varied with the tested variant allele from 0.100 (rs7498665 *SH2B1*) to 0.309 (rs6020712 *CTNNBL1*).

### Variants associated with BMI z-score in SGA children

Ten variants in seven genes were significantly associated with BMI z-score in SGA children after correction for multiple testing (Table 2). The impact on BMI z-score increase varied from 0.133 (rs2815752 *NEGR1*) to 0.458 (rs6020395 *CTNNBL1*). In contrast to those born AGA, none of the 5 variants in MC4R were associated with increasing BMI z-score in those born SGA.

### Variants associated with BMI z-score after adjustment for SGA status

A total of 20 variants: rs2286385 (*ADIPOR2*), rs6265 (*BDNF*), rs16986921, rs6013029, rs6020395, rs6020712, rs6020846, rs6096781, and rs6125962 (*CTNNBL1*), rs6499640, rs8050136, and rs9939609 (*FTO*), rs1042725 (*HMGA2*), rs11084753 (*KCTD15*), rs12970134, rs17782313, rs4450508, and rs502933 (*MC4R*), rs10508503 (*PTER*), and rs10913469 (*SEC16B*) showed significant association after adjustment SGA status and also correction for multiple testing. BMI z-score increased with all tested obesity risk variant alleles ranging from 0.087 (rs1042725 *HMGA2*) to 0.303 (rs6013029 *CTNNBL1*).

### Variants associated with interaction between BMI z-score and SGA status

A total of eight variants: rs1424233 (*MAF*), rs12970134 and rs17782313, (*MC4R*), rs2293855 (*MTMR9*), rs2568958 and rs2815752 (*NEGR1*), rs10508503 (*PTER*), and rs7498665 (*SH2B1*) showed significant interaction with SGA status. Two of these variant (*MC4R* and *PTER*) also showed a significant association with BMI z-score among the whole cohort with adjustment for SGA status, however, their effect was only observed among those born AGA, with no effect in SGA. Of the remaining obesity risk variants showing a significant interaction with SGA status, two variants in *NEGR1* were associated with increased BMI in those born SGA but no effect in those born AGA, while *SH2B1* obesity risk variant showed increased BMI in those born SGA and reduced BMI in those born AGA. There was a significant interaction between the *MAF* variant and SGA status, and reduced BMI in the SGA group (Table 2). However, there were no significant effects on BMI when the association was tested within those born SGA and AGA.

## Discussion

We confirmed 27 variants within 14 obesity genes to be individually associated with increasing early childhood BMI in those born AGA, most of which were also associated with increasing early childhood BMI in the whole cohort after adjusting for SGA status. Variants associated with the interaction of BMI z-score and SGA status were largely driven by their predominant association with increasing BMI in either the AGA group (*MC4R, PTER, MTMR9*), or the SGA group (*NEGR1, SH2B1*), rather than a consistently larger effect seen in the SGA group as hypothesised. None of the 5 *MC4R* obesity risk variants associated with increasing BMI in AGA were significant in those born SGA. However, all three of the *FTO* obesity risk variants showed an effect of increasing BMI in those born SGA, one of which was also significant in those born AGA.

Although the biological functions of the majority of the obesity gene variants are still being investigated, most of the obesity risk variants are thought to act centrally on increasing hyperphagic drive [19]. The other physiological mechanisms by which these gene variants could be acting to influence postnatal growth would be through a change in control of body’s energy partitioning from lean to fat tissue, or shift in metabolic efficiency through thermogenesis.

Our New Zealand SGA birth cohort did not show evidence of accelerated postnatal growth, in terms of their BMI z-score, which remained lower than those born AGA at assessment age 3.5, 7 and 11 years, however, they may still have increased postnatal fat deposition relative to lean body or skeletal mass. There is evidence from studies conducted in Switzerland [20] and Spain [21] that pre-pubertal children born SGA have more body fat and less lean tissue than weight or age matched controls. Nonetheless, the higher cardiovascular risks observed with accelerated postnatal growth in those born SGA have been demonstrated to occur with a rapid tempo of childhood gain in BMI rather than body fat [22].

Our results do not suggest that the known obesity risk gene variants have a disproportionately higher impact on increasing childhood BMI in those born SGA compared to AGA. However, until the age of 11 years, those born SGA remained smaller than AGA children, and it remains to be seen whether they develop a greater prevalence of obesity in adulthood. This work leads us to believe that non-genetic or environmental factors are more likely to be important in influencing childhood BMI in those born SGA.

## Conclusions

In summary, our longitudinal data with measurement of BMI at 3.5, 7 and 11 years of age, confirm that several of the adult obesity risk gene variants (*ADIPOR2, BCDIN3D_FAIM2, BDNF, CTNNBL1, FTO, HMGA2 KCTD15, MTMR9 MC4R, PTER, SEC16B,* and *SH2B1)* were also associated with childhood growth in New Zealand. Most of the association between obesity variants with childhood BMI was observed within children born AGA rather than the SGA, suggesting that non-genetic or environmental factors may be more important in influencing childhood BMI in those born SGA.

## Abbreviations

ABC: Auckland Birthweight Collaborative; AGA: Appropriate-for-gestational-age; SGA: Small-for-gestational-age; SNPs: Single nucleotide polymorphisms; CEPH: Centre d’Etude du Polymorphisme Humain; HWE: Hardy Weinberg Equilibrium; MALDI-TOF: Matrix Assisted Laser Desorption/Ionization – Time of Flight.

## Competing interests

The authors declare that they have no competing interests.

## Authors’ contributions

DYH performed the data analyses. DYH and RM wrote the manuscript. ARM and WJL performed genotyping and contributed to the writing of the manuscript. LRF, CRW, and KEW contributed to the writing of the manuscript. JMDT had responsibility for outcome assessment and contributed to the writing of the manuscript. EAM supervised the design and execution of the study and contributed to the writing of the manuscript. All authors read and approved the final manuscript.

## Pre-publication history

The pre-publication history for this paper can be accessed here:

http://www.biomedcentral.com/1471-2350/14/10/prepub
